# The Effects of Tea Wastes Prepared Using Different Composting Methods on the Seedling Growth and Selected Biochemical Properties of Maize (*Zea mays* var. *indurata*)

**DOI:** 10.1002/fsn3.70670

**Published:** 2025-07-30

**Authors:** Gözde Hafize Yıldırım, Ebru Batı Ay, Mustafa Doğukan Şahin

**Affiliations:** ^1^ Department of Field Crops, Faculty of Agriculture, Recep Tayyip Erdoğan University Rize Turkey; ^2^ Department of Plant and Animal Production, Suluova Vocational School, Amasya University Amasya Turkey

**Keywords:** ascorbate peroxidase 1, flavonoid 2, local maize genotypes 5, proline 3, tea compost 4 sustainable agriculture 5

## Abstract

Maize is a vital cereal crop globally and a key component of Turkey's agriculture. Sustainable practices, particularly the use of organic waste, are crucial for maintaining soil health and reducing environmental impact. Tea waste, rich in organic matter and bioactive compounds, presents a promising resource for composting and supports the circular economy. This study aimed to evaluate the effects of tea waste‐based composts, prepared using different composting methods, on seedling growth and selected biochemical properties of maize. Five treatment combinations were established using tea composts enriched with various organic fertilizers, including chicken manure, vermicompost, and bat guano. The treatments were as follows: tea compost+chicken manure (TC+CM), tea compost+chicken manure+bat guano (TC+CM+BG), tea compost+bat guano (TC+BG), tea compost+bat guano+vermicompost (TC+BG+VC), and tea compost+vermicompost (TC+VC). To allow a broader evaluation of treatment effects, three local maize genotypes (G1, G2, G3) were included in the experiment. The effects of compost treatments were evaluated at the seedling stage based on key biochemical and morphological parameters. Different compost applications led to substantial differences in the biochemical and morphological characteristics of maize seedlings. The highest total phenolic content was observed in G1–TC+BG+VC; total flavonoid accumulation in G2–TC+VC and proline accumulation in G3–TC+VC; total antioxidant capacity in G2–TC+CM+BG; catalase activity in G1–TC and G3–TC+BG; ascorbate peroxidase activity in G3–TC+BG+VC; total chlorophyll in G3–TC+CM; and total carotenoid in G3–TC+BG treatments. Morphologically, the tallest seedlings were obtained in G1–TC+VC, the largest leaf area in G3–TC, and the highest fresh seedling weight in G3–TC+CM treatments. Enhanced tea compost mixtures positively influenced various biochemical and morphological traits in maize seedlings. Notably, the TC+CM, TC+VC, and TC+BG+VC treatments outperformed others across multiple parameters, demonstrating their potential to improve plant growth. These findings offer a promising pathway toward more environmentally conscious agricultural practices and reinforce the prospects of sustainable crop production.

## Graphical abstract

This study investigates the effects of tea‐waste‐based composts enriched with various organic fertilizers on the seedling growth and biochemical responses of local maize genotypes. Compost mixtures, especially those containing chicken manure and vermicompost, significantly enhanced plant development and antioxidant activity. The findings highlight the potential of tea waste as a sustainable alternative to chemical fertilizers in maize cultivation.

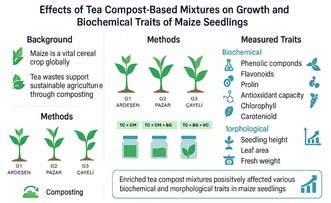

## 1 Introduction

Maize ( *Zea mays* L.) is one of the most widely cultivated and consumed cereal crops worldwide, with annual production exceeding 1 billion tons (Galindo et al. 2024; Erenstein et al. 2022). In maize agriculture, various scientific and technological strategies have been developed to reduce the excessive dependence on chemical fertilizers and to address the associated environmental problems (Agiragac and Çelebi 2024). These strategies aim to ensure the sustainability of agricultural production while minimizing environmental degradation. In particular, it is crucial to implement agricultural practices that increase productivity without harming ecosystems and that conserve natural resources (Galindo et al. 2019).

Organic and biofertilizers contribute to the development of healthy and productive agricultural systems by enhancing soil microbial diversity, improving soil structure, and supporting nutrient uptake by plants. These fertilizers enrich the soil ecosystem, support plant growth, and promote the adoption of sustainable agricultural practices (Çiftçi et al. 2024; Kour et al. 2020; Dasgupta et al. 2021; Gautam et al. 2021; Villamarin‐Raad et al. 2023). Solid or liquid fertilizers derived from tea compost enhance biological activity in the soil due to their content of beneficial microorganisms, microbial metabolites, and essential nutrients (Scheuerell and Mahaffee 2002). These effects contribute to the improvement of soil quality and support plant development (Li et al. 2018). The effectiveness of such composts is largely associated with the compounds released or activated during fermentation. Moreover, conditions such as temperature, duration, and aeration during the fermentation process directly influence the nutrient content and microbial structure of tea composts (Islam et al. 2016). Enriching tea compost with various organic fertilizers can further enhance its nutrient composition and increase both the magnitude and duration of its beneficial effects.

### 1.1 The Effects of Organic Waste Composting on Soil Fertility and Plant Productivity

Emerging approaches to the use of organic amendments in agriculture are increasingly recognized as effective strategies for improving soil structure, enhancing productivity, and promoting crop quality. Composting is a natural process in which organic materials decompose biologically—primarily under aerobic conditions (Bernal et al. 2009). During composting of organic waste, simple organic carbon compounds are readily mineralized and metabolized by microorganisms, releasing CO_2_, NH_3_, H_2_O, organic acids, and heat. This biological decomposition provides substantial benefits for improving soil quality and supporting plant nutrition (Lee et al. 2004).

Composted organic residues contain essential macro and micronutrients required for plant development, such as nitrogen, phosphorus, potassium, magnesium, iron, and zinc. Through this, they help reduce the need for chemical fertilizers while contributing to the maintenance of soil health (Palansooriya et al. 2023; Litskas 2023). In particular, organic fertilizers such as vermicompost and food waste compost (FOWC) have been shown to enhance soil microbial activity, enzymatic processes, and water‐holding capacity—thereby exerting positive effects on plant growth (Hazra 2016; Chen et al. 2018; Arancon et al. 2003; Kang et al. 2021; Patel et al. 2021). Organic matter serves as an excellent source of plant‐available nutrients, and its addition to soil supports the persistence of high microbial populations and activity (Arancon and Edwards 2005). By improving water retention and aeration, organic matter enhances the physical properties of soil, prevents surface crusting, and supports the development of soil structure—ultimately creating a favorable environment for plant growth (Taban et al. 2013; Guo et al. 2019; Yaylacı and Erdal 2021).

When applied to soil in specific proportions, organic wastes have been shown to directly promote plant development. Particularly, thermally pre‐treated composts, conventional composts, and pre‐processed food waste materials are considered effective organic fertilizer alternatives that support plant growth (Han et al. 2024). Although compost alone may not fulfill the complete nutrient requirements of plants, its high organic matter content and capacity to enhance biological activity can significantly improve mineral nutrition, crop yield, and product quality (Atiyeh et al. 2000; Gutiérrez‐Miceli et al. 2007; Joshiand and Vig 2010). During the decomposition of organic matter, humic substances, hormone‐like compounds, vitamins, and enzymes are released, which promote both root development and aboveground biomass formation (Erdal and Ekinci 2020; Yaylacı et al. 2024).

### 1.2 Different Components of Composts and the Role of Tea Waste as a Fertilizer

Animal‐based manures and plant‐derived composts contain essential macro (N, P, K, Ca, Mg) and micro (Fe, Mn, Zn) elements at varying levels, which are critical for plant nutrition and soil fertility (Aygün and Acar 2004). For instance, sheep manure contains 2.0% N, 0.10% P, 0.18% K, 1.16% Ca, and 0.13% Mg, whereas goat manure is notable for its 1.9% N, 0.08% P, 0.80% K, 0.009% Ca, and 0.015% Mg content. Quail manure stands out for its relatively high phosphorus (1.12%) and potassium (1.18%) levels, in addition to 2.3% N, 0.05% Ca, and 0.50% Mg. Similarly, pigeon manure provides high nutrient content with 3.5% N, 0.64% P, and 1.04% K. Bat guano, with its 7.3% nitrogen content, represents one of the richest nitrogen sources and is also noteworthy for its substantial micronutrient levels, including 780 ppm Zn and 490 ppm Mn. Among plant‐based compost materials, rose compost contains 4.0% N and 623 ppm Fe; kitchen waste compost is particularly rich in nutrients, with 3.1% N, 1.24% K, 4.27% Ca, 0.68% Mg, 6357 ppm Fe, 130 ppm Mn, and 162 ppm Zn. Likewise, mushroom compost emerges as a highly enriched organic material with 2.2% N, 1.66% K, 4.11% Ca, 1.24% Mg, 6778 ppm Fe, 240 ppm Mn, and 193 ppm Zn (Demirtaş et al. 2005).

Waste generated by tea processing factories—particularly lignocellulosic organic materials widespread in the Black Sea Region—possess high organic matter content, rendering them suitable for compost production (Gürten 2008; Altay 2021). Despite their regional abundance, detailed composting methodologies for these materials are scarcely discussed in the literature and remain underutilized in agricultural practice. The organic carbon content of tea waste piles varies with the composting duration. Rakıcıoğlu and Kızılkaya (2021) reported that a compost pile initially containing 51.4% organic carbon retained a similar level (51.8%) by the end of the composting process. Moreover, the final compost exhibited favorable C/N ratios and pH values, indicating its suitability for agricultural soils. This compost can also be prepared in liquid form and applied widely in agriculture.

Compost tea, a biologically active liquid organic fertilizer obtained through fermentation of mature compost in an aqueous medium, enhances soil fertility by enriching microbial activity through its content of macro‐ and micronutrients, beneficial microorganisms, and their metabolites (Scheuerell and Mahaffee 2002). This liquid formulation can be applied to both soil and foliage and has shown efficacy in suppressing fungal diseases such as mold, wilt, root rot, and powdery mildew (Litterick et al. 2004; Morales‐Corts et al. 2018). Additionally, it can be applied directly to the root zone via drip irrigation systems or used as a nutrient solution in soilless cultivation systems (Ros et al. 2020; Shaban et al. 2015; Wu et al. 2020). Valorization of regionally abundant organic residues such as tea waste through this method not only offers an advantage in waste management but also contributes to reducing dependency on synthetic inputs, aligning with the principles of the circular economy (Yin et al. 2025). From a circular economy perspective, tea waste–based compost production enables the reutilization of an underutilized biomass resource. This process not only minimizes the need for synthetic inputs but also promotes soil health, thereby supporting sustainable agricultural practices (De Corato 2020). In this context, the main objective of the present study is to comparatively evaluate the effects of compost formulations enriched with tea waste and various organic fertilizers (bat guano, vermicompost, and poultry manure) on seedling growth performance and specific biochemical traits in three local maize ( *Zea mays* L. var. *indurata*) genotypes.

## 2 Materials and Methods

This study comparatively evaluated the effects of tea composts enriched with organic fertilizers on the biochemical properties and seedling growth parameters of three different maize ( *Zea mays* var. *indurata* ) genotypes. Five different fertilizer composts and three maize genotypes were used in the study. The characteristics of the maize genotypes used are as follows: Genotype 1 (G1) is the local yellow‐white mixed kernel maize collected from the Ardeşen district of Rize (Turkey); Genotype 2 (G2) is the local yellow‐white mixed kernel maize obtained from the Pazar district of Rize; and Genotype 3 (G3) is the local yellow‐white mixed kernel maize sourced from the Çayeli district of Rize. The fertilizer applications used in the study were prepared in different mixtures. The first application contains a mixture of Tea Compost (TC) (13,070 mL) and Chicken Manure (CM) (200 mL). The second application consists of Tea Compost (TC) (13,070 mL), Chicken Manure (CM) (200 mL), and Bat Guano (BG) (200 mL). The third application contains a mixture of Tea Compost (TC) (13,070 mL) and Bat Guano (BG) (200 mL). The fourth application encompasses a mixture of Tea Compost (TC) (13,070 mL), Bat Guano (BG) (200 mL), and Worm Compost (WC) (200 mL). The fifth application includes a mixture of Tea Compost (TC) (13,070 mL) and Worm Compost (WC) (200 mL). The plants were cultivated in plastic pots (16 cm in diameter, 13 cm in depth) filled with a sterile peat medium (pH 6.0, supplemented with 1.0 g/L fertilizer). A factorial experimental design with three replications was employed.

The organic fertilizer contents used in the study are as follows: Bat Guano: Bat guano is a solid organic fertilizer containing 25% organic matter, 7% organic carbon, and 2% total organic nitrogen (w/w, weight/weight). It also includes 5% water‐soluble potassium oxide (K_2_O), 1% total phosphorus pentoxide (P_2_O_5_), 3% humic and fulvic acids, and 1% water‐soluble calcium oxide (CaO). The pH (potential of hydrogen) range is between 5.0 and 7.0, and the maximum electrical conductivity (EC) is reported as 17 dS/m (deciSiemens per meter). Organic Liquid Worm Compost: This liquid vermicompost extract contains 7% organic matter and 0.3% organic nitrogen (w/w). Its maximum electrical conductivity (EC) is 0.5 dS/m, with a pH range of 4.5 to 6.5. Chicken Manure Extract: The chicken manure extract used in this study contains 10% total nitrogen and 40% organic matter (w/w). Tea Waste Compost: Tea waste compost was obtained from a local tea processing factory. It contains 42.03% organic carbon (w/w), has a pH value of 5.72, and an electrical conductivity (EC) of 2.18 dS/m.

The prepared mixtures were blended until a homogeneous structure was achieved and then left to ferment under anaerobic conditions in a plastic bag at 25°C–30°C for 50 days. After the fermentation process was completed, the bag was opened, and the mixture was exposed to oxygen for 1 day before application. The prepared fertilizer mixtures were applied to the soil 60 days after sowing, and the plants were harvested after 90 days. Ten days after the application, specific parts of the plants were harvested for biochemical analyses. Agronomic measurements were performed 20 days later to allow for more pronounced morphological changes. Fresh plant samples were stored at 4°C until the analyses were performed.

## 3 Biochemical Analyses Performed

Total Phenolic Content (mg GAE/g): Collected leaf samples were extracted with methanol and mixed with Folin–Ciocalteu reagent and sodium carbonate solution. The mixture was incubated in the dark for 1 h and measured at 765 nm. The results are expressed as gallic acid equivalent (GAE) (Siddhuraju and Manian 2007).

Total Flavonoid Content (mg QE/g): Collected leaf samples were incubated after the addition of NaNO_2_ and AlCl_3_. Total flavonoid content was measured at 510 nm using a quercetin standard, and the results are expressed as quercetin equivalents (QE) (Zhishen et al. 1999).

Total Antioxidant Content (%): Collected leaf samples were measured using the DPPH radical scavenging assay. 0.1 mL of the sample was mixed with 1 mL DPPH solution and 4 mL methanol, then incubated in the dark for 30 min. Absorbance was measured at 517 nm, and the scavenging activity was calculated (Brand‐Williams et al. 1995).

Proline (μmol/mL): Proline analysis was performed using the Bates (Bates 1973) method. Leaf tissue (50 mg) was homogenized with a 5 mL solution containing 3% sulfosalicylic acid and centrifuged at 5000 g for 10 min. The obtained supernatant was mixed with 2 mL acid‐ninhydrin and 2 mL acetic acid and incubated at 100°C for 1 h. The samples were then cooled and extracted with 5 mL toluene. The absorbance of the upper phase was measured at 520 nm, and the proline amount was calculated using a standard curve, expressed in μmol/g fresh weight.

Catalase (CAT) (EU/mL): Catalase activity was analyzed using the Cho (Cho et al. 2000) method. The reaction mixture contained phosphate buffer, H₂O₂ (hydrogen peroxide), and water and was incubated at 30°C for 3 min. A quartz cuvette was used to measure absorbance at 240 nm, and catalase activity was calculated using the formula ΔAbs (240 nm)/(minute × milligram protein), expressed as EU/mg protein.

Askorbat Peroksidaz (APX) (EU/mL) (enzyme unit per milliliter): APX activity was determined using the Nakano (Nakano and Asada 1981) method. Leaf tissue (0.5 g) was homogenized at +4°C in an extraction buffer (0.1 mM EDTA ethylenediaminetetraacetic acid), 2 mM ascorbate, 2% PVPP (polyvinylpolypyrrolidone), 50 mM PBS (phosphate‐buffered saline), pH 7 and centrifuged at 14,000 rpm (revolutions per minute) for 30 min. The obtained supernatant was used for APX activity measurements, and the activity was calculated using ΔAbs (290 nm)/(min × mg protein), expressed as U/mg protein.

Pigment Analyses: Total chlorophyll and carotenoid content were analyzed using the Arnon (Arnon 1949; Yildirim and Ay 2023) method, and chlorophyll a, chlorophyll b, total chlorophyll, and total carotenoid amounts were calculated using the following formulas:

$$\text{Chlorophyll}\;a\;\left(\mathrm{Ca}\right)=\left[12.7\times \Delta 663-2.69\times \Delta 645\right]\times \mathrm{mL}/100$$

$$\text{Chlorophyll}\;b\;\left(\mathrm{Cb}\right)=\left[22.9\times \Delta 645-4.68\times \Delta 663\right]\times \mathrm{mL}/1000$$

$$\text{Total Chlorophyll}\;\left(C\right)=\left[20.2\times \Delta 645+8.02\times \Delta 663\right]\times \mathrm{mL}/1000$$

$$\begin{array}{c} \text{Total Carotenoid}\\ =\left[1000\;\Delta 470-1.90\times \mathrm{Ca}-63.14\times \mathrm{Cb}/214\right]\times \mathrm{mL}/1000 \end{array}$$

Note: Absorbance measurements at 645 nm (nanometer), 663 nm, and 470 nm wavelengths were performed using a spectrophotometer.

Spectrophotometric analyses were performed using the Evolution 201 model spectrophotometer by Thermo Scientific. The spectrophotometer was manufactured by Thermo Fisher Scientific Inc. (Address: 168 Third Avenue, Waltham, MA 02451, USA; Website: https://www.thermofisher.com).

## 4 Statistical Analysis

Statistical analyses were performed using JMP (John's Macintosh Project) software. Data were evaluated using two‐way analysis of variance (Two‐way ANOVA) (Kiremit et al. 2023), and differences between groups were analyzed using Tukey's multiple comparison test. JMP software is developed by SAS Institute Inc. (Address: SAS Campus Drive, Cary, NC 27513, USA; Website: https://www.jmp.com).

## 5 Results

### 5.1 Total Phenolic and Flavonoid Content

Statistically significant effects of different genotypes and treatments on total phenolic content (TPC) were observed (*p* ≤ 0.001). Among the genotypes, the highest TPC value was recorded in G2 (194.74 ± 5.85 mg GAE/g DW), followed by G1 (181.98 ± 5.85) and G3 (167.77 ± 5.85). Regarding treatments, the highest TPC was measured in the TC+CM application (194.77 ± 8.28 mg GAE/g DW), followed by TC+BG+VC (192.17 ± 8.28) and TC+VC (187.82 ± 8.28), while the lowest value was found in the TC treatment (158.15 ± 8.28) (Table 1).

**TABLE 1:** Mean values and significance levels of phenolic content, flavonoids, and antioxidant capacity affected by different genotypes and applications.

Genotype		TPC	Genotype		TFC	Genotype		TAC
G2	a	194.74 ± 5.85	G2	a	136.47 ± 0.28	G3	a	95.80 ± 0.07
G1	ab	181.98 ± 5.85	G1	b	125.77 ± 0.28	G2	b	94.89 ± 0.07
G3	b	167.77 ± 5.85	G3	c	109.77 ± 0.28	G1	c	88.70 ± 0.07
CV (%)		9.67	CV (%)		0.94	CV (%)		0.30
TPC		***	TFC		***	TAC		***

Applications		TPC	Applications		TFC	Applications		TAC
TC+CM	a	194.77 ± 8.28	TC+BG+VC	a	138.83 ± 0.55	TC+VC	a	94.76 ± 0.13
TC+BG+VC	a	192.17 ± 8.28	TC+BG	b	134.56 ± 0.55	TC+BG	b	93.67 ± 0.13
TC+VC	a	187.82 ± 8.28	TC+VC	c	129.42 ± 0.55	TC	c	93.06 ± 0.13
TC+CM+BG	a	186.58 ± 8.28	TC+CM	d	118.52 ± 0.55	TC+CM+BG	c	92.94 ± 0.13
TC+BG	ab	180.02 ± 8.28	TC+CM+BG	e	116.09 ± 0.55	TC+CM	c	92.64 ± 0.13
TC	b	158.15 ± 8.28	TC+BG+VC	a	106.60 ± 0.55	TC+BG+VC	d	91.59 ± 0.13
CV (%)		9.67	CV (%)		0.94	CV (%)		0.30
TPC		***	TFC		***	TAC		***

Abbreviations: BG, Bat Guano; CM, Chicken Manure; CV, Coefficient of Variation Value; G1, G2, G3, Genotype 1, Genotype 2, Genotype 3; N.S., not significant; TAC, Total Antioxidant Content (25 μg/mL); TC, Tea Compost; TFC, Total Flavonoid Content (mg Quercetin Equivalent/g Dry Weight); VC, Vermicompost.

*** *p* ≤ 0.001.

Total flavonoid content (TFC) was also significantly affected by both genotype and treatment (*p* ≤ 0.001). The highest TFC was found in genotype G2 (136.47 ± 0.28 mg QE/g DW), which differed significantly from G1 (125.77 ± 0.28) and G3 (109.77 ± 0.28). Among the treatments, the TC+BG+VC application yielded the highest TFC value (138.83 ± 0.55 mg QE/g DW), followed by TC+BG (134.56 ± 0.55) and TC+VC (129.42 ± 0.55). The lowest flavonoid content was again observed in the TC+BG+VC treatment (106.60 ± 0.55) (Table 1).

**FIGURE 1:**
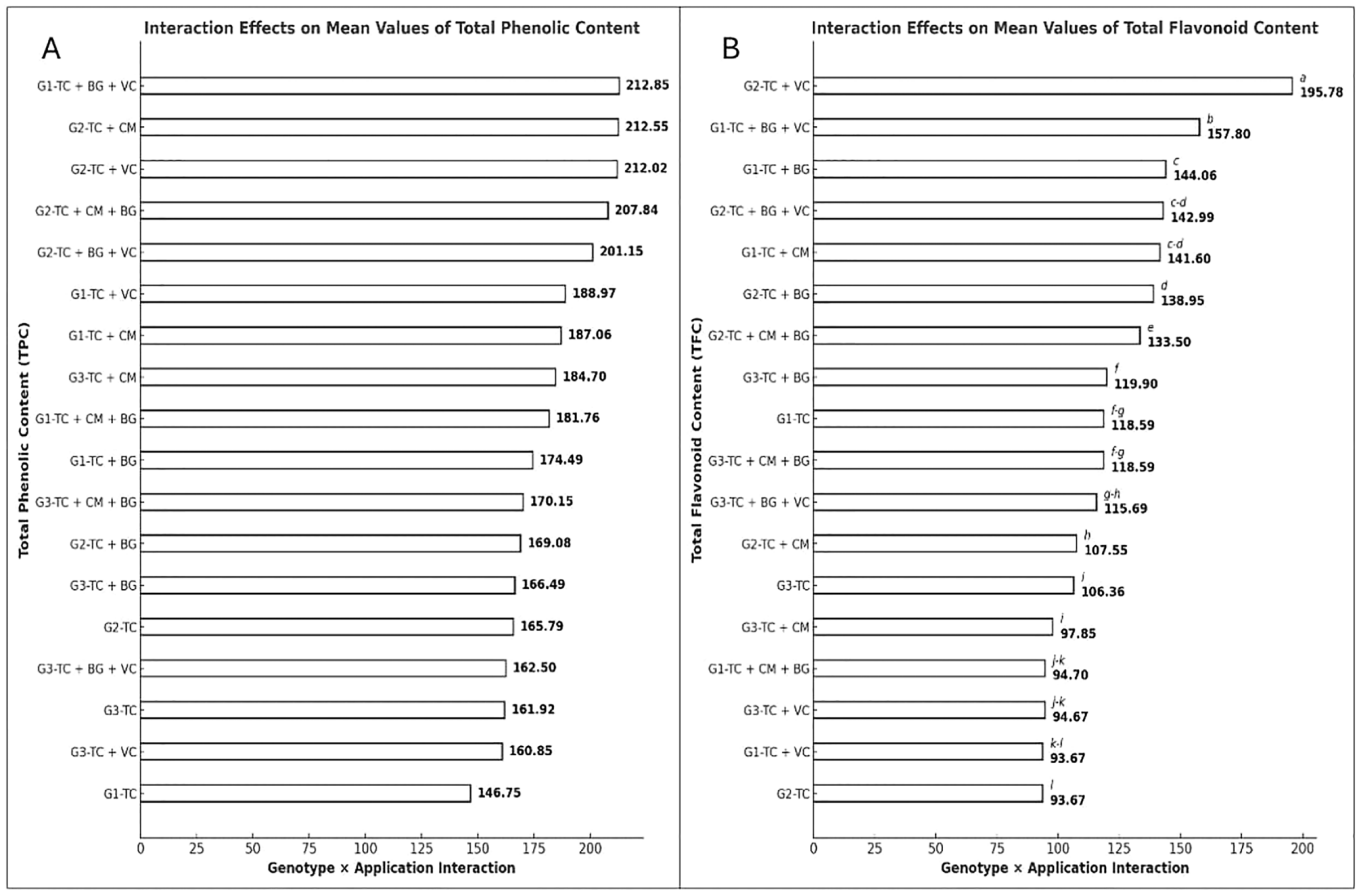
(A) Effects of genotype × treatment interactions on total phenolic content (TPC) (mg GAE/g DW). (B) Effects of genotype × treatment interactions on total flavonoid content (TFC) (mg QE/g DW). Different letters indicate statistically significant differences between group means (Tukey's test, *p* ≤ 0.001). Groups sharing the same letter are not significantly different; groups without letters show no statistically significant differences. BG, Bat Guano; CM, Chicken Manure; G1, G2, G3, Genotype 1, Genotype 2, Genotype 3; TC, Tea Compost; VC, Vermicompost.

Regarding genotype **×** treatment interactions, no statistically significant differences were found for TPC (Figure 1A). However, significant differences were detected in TFC levels among the genotype **×** treatment combinations (*p* ≤ 0.001) (Figure 1A). The highest TFC value was observed in the G2–TC+VC combination (195.78 ± 0.96 mg QE/g DW), which was statistically distinct from all other combinations. This was followed by G1–TC+BG+VC (157.80 ± 0.96), G1–TC+BG (144.06 ± 0.96), and G2–TC+BG+VC (142.99 ± 0.96). The lowest TFC values were recorded in G2–TC (93.67 ± 0.96), G1–TC+VC, G3–TC+VC, and G1–TC+CM+BG, falling within the 94.70–93.67 mg QE/g DW range (Figure 1B).

### 5.2 Total Antioxidant Capacity and Proline Content

Total antioxidant capacity (TAC) was significantly influenced by both genotype and treatment factors (*p* ≤ 0.001). Among the genotypes, G3 exhibited the highest TAC value (95.80 ± 0.07 mg TE/g DW), followed by G2 (94.89 ± 0.07) and G1 (88.70 ± 0.07). In terms of treatments, the highest TAC was recorded under the TC+VC application (94.76 ± 0.13 mg TE/g DW), followed by TC+BG (93.67 ± 0.13) and TC (93.06 ± 0.13). The lowest antioxidant capacity was observed in the TC+BG+VC treatment (91.59 ± 0.13) (Table 1).

**FIGURE 2:**
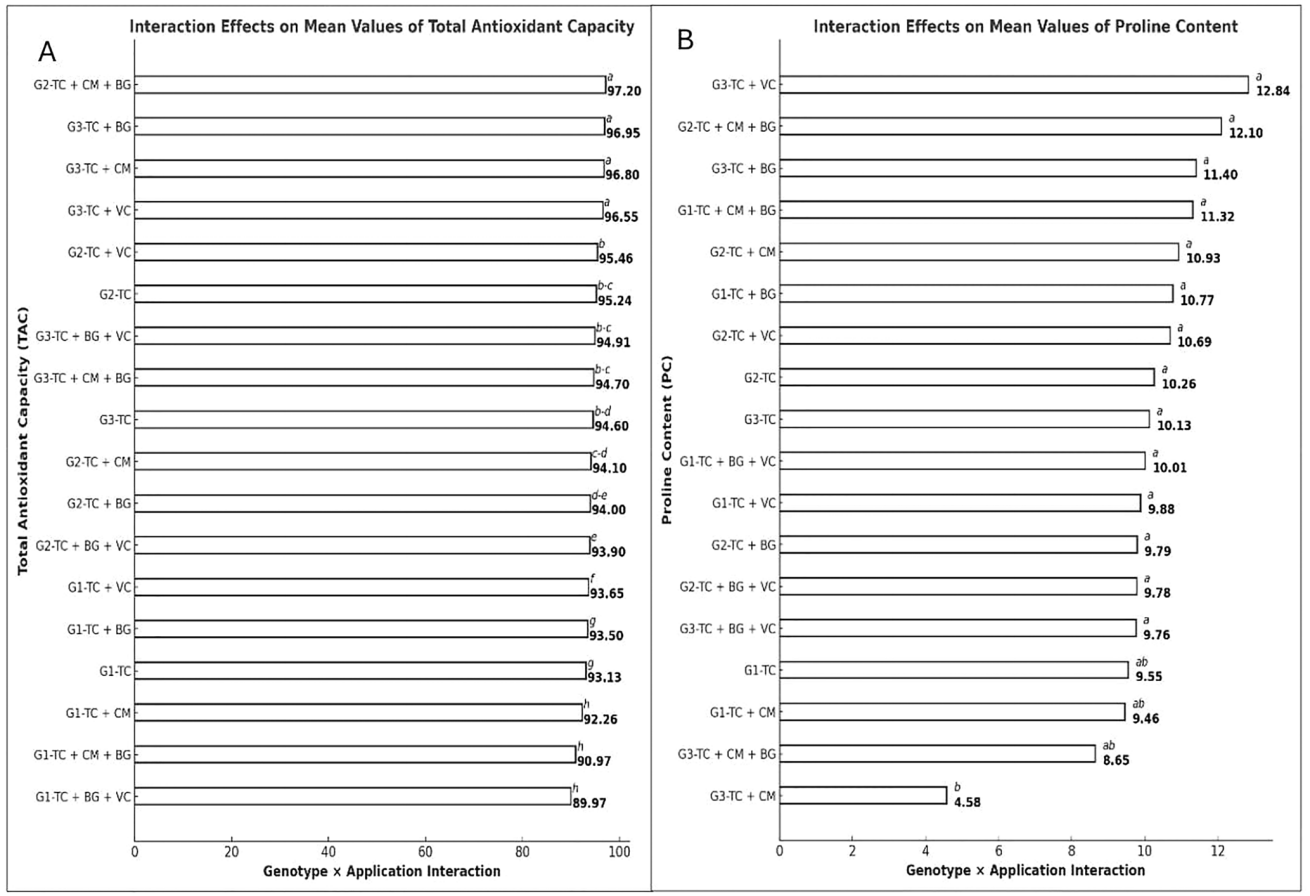
(A) Effects of genotype × treatment interactions on total antioxidant capacity (TAC) (unitless). (B) Effects of genotype × treatment interactions on proline content (PC) (μmol/g DW). Different letters indicate statistically significant differences among group means according to Tukey's test (*p* ≤ 0.001). Groups sharing the same letter are not significantly different; groups without letters show no statistically significant difference. BG, Bat Guano; CM, Chicken Manure; G1, G2, G3, Genotype 1, Genotype 2, Genotype 3; TC, Tea Compost; VC, Vermicompost.

A statistically significant genotype × treatment interaction was also detected for TAC levels (*p* ≤ 0.001). The highest TAC was observed in the G2–TC+CM+BG combination (97.20 ± 0.23 mg TE/g DW), followed by G3–TC+BG (96.95 ± 0.23) and G3–TC+CM (96.80 ± 0.23). Conversely, the lowest TAC was recorded in the G1–TC+BG+VC combination (89.97 ± 0.23), suggesting that this specific treatment may exert a limited effect on antioxidant activity (Figure 2A).

Regarding proline content, genotype alone did not have a statistically significant effect (*p* > 0.05). However, considering the numerical differences, G2 exhibited the highest proline accumulation (10.59 ± 0.39 μg/μL), followed by G1 (10.16 ± 0.39) and G3 (9.56 ± 0.39). On the other hand, proline content was significantly affected by treatment (*p* ≤ 0.05). The highest levels were observed under TC+VC (11.13 ± 0.78 μg/μL) and TC+CM+BG (10.69 ± 0.78) applications, whereas the lowest proline accumulation occurred under the TC+CM treatment (8.32 ± 0.78) (Table 2).

**TABLE 2:** Mean values and significance levels of proline, catalase, and ascorbate peroxidase affected by different genotypes and applications.

Genotype		PC	Genotype		CAT	Genotype		APX
*G2*	—	10.59 ± 0.39	*G3*	a	2.37 ± 0.01	*G3*	a	0.15 ± 0.01
*G1*	—	10.16 ± 0.39	*G1*	b	2.17 ± 0.01	*G2*	b	0.07 ± 0.01
*G3*	—	9.56 ± 0.39	*G2*	c	1.33 ± 0.01	*G1*	c	0.05 ± 0.01
CV (%)		16.33	CV (%)		0.51	CV (%)		11.18
*PC*		N.S.	*CAT*		***	*APX*		***

Applications		PC	Applications		CAT	Applications		APX
*TC*+*VC*	a	11.13 ± 0.78	*TC*+*BG*	a	3.47 ± 0.01	*TC*+*BG*+*VC*	a	0.19 ± 0.01
*TC*+*CM*+*BG*	a	10.69 ± 0.78	*TC*	b	2.40 ± 0.01	*TC*+*BG*	b	0.07 ± 0.01
*TC*+*BG*	ab	10.65 ± 0.78	*TC*+*VC*	c	2.37 ± 0.01	*TC*+*CM*	c	0.07 ± 0.01
*TC*	ab	9.98 ± 0.78	*TC*+*BG*+*VC*	d	1.83 ± 0.01	*TC*	c‐d	0.07 ± 0.01
*TC*+*BG*+*VC*	ab	9.85 ± 0.78	*TC*+*CM*	e	1.57 ± 0.01	*TC*+*VC*	d‐e	0.06 ± 0.01
*TC*+*CM*	b	8.32 ± 0.78	*TC*+*CM*+*BG*	f	1.07 ± 0.01	*TC*+*CM*+*BG*	e	0.05 ± 0.01
CV (%)		16.33	CV (%)		0.51	CV (%)		11.18
*PC*		**	*CAT*		***	*APX*		***

Abbreviations: APX, Ascorbate Peroxidase Activity (EU/ml) (25 μL—290 nm); BG, Bat Guano; CAT, Catalase Activity (EU/mL) (50 μL—240 nm); CM, Chicken Manure; CV, Coefficient of Variation Value; G1, G2, G3, Genotype 1, Genotype 2, Genotype 3; N.S., not significant; PC, Proline Content (μg/mL); TC, Tea Compost; VC, Vermicompost.

** *p* ≤ 0.01.

*** *p* ≤ 0.001.

A highly significant genotype × treatment interaction was also found for proline content (*p* ≤ 0.001). The G3–TC+VC combination resulted in the highest proline concentration (12.84 ± 1.35 μg/μL), followed by G2–TC+CM+BG (12.10 ± 1.35) and G3–TC+BG (11.40 ± 1.35). Other notable combinations included G1–TC+CM+BG (11.32 ± 1.35) and G2–TC+CM (10.93 ± 1.35), while the lowest value was recorded in G3–TC+CM (4.58 ± 1.35) (Figure 2B).

### 5.3 Catalase and Ascorbate Peroxidase Activities

Catalase (CAT) activity was significantly affected by both genotype and treatment factors (*p* ≤ 0.001). Among the genotypes, the highest CAT activity was recorded in G3 (2.37 ± 0.01 EU/mL), followed by G1 (2.17 ± 0.01) and G2 (1.33 ± 0.01). G3 exhibited a significantly higher enzyme activity compared to the other genotypes. Regarding the treatments, the highest CAT activity was observed in the TC+BG application (3.47 ± 0.01 EU/mL), whereas the lowest activity was found in the TC+CM+BG treatment (1.07 ± 0.01) (Table 2).

**FIGURE 3:**
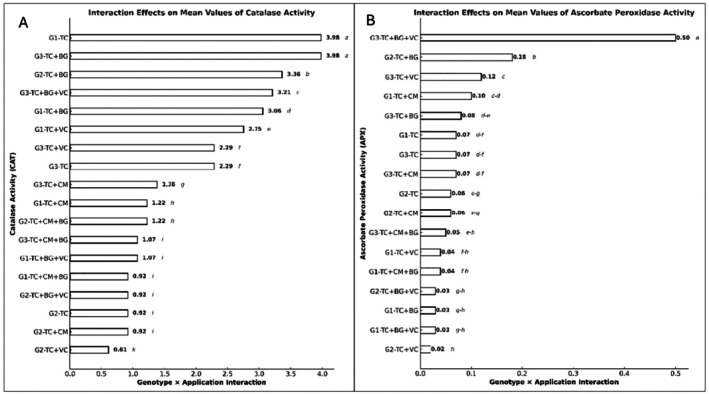
(A) Effects of genotype × treatment interactions on catalase (CAT) activity (U/mg protein). (B) Effects of genotype × treatment interactions on ascorbate peroxidase (APX) activity (U/mg protein). Different letters indicate statistically significant differences among group means according to Tukey's test (*p* ≤ 0.001). Groups sharing the same letter are not significantly different; groups without letters show no statistically significant difference. BG, Bat guano; CM, Chicken manure; G1, G2, G3, Genotype 1, Genotype 2, Genotype 3; TC, Tea compost; VC, Vermicompost.

A statistically significant genotype × treatment interaction was also observed for catalase activity (*p* ≤ 0.001). The highest values were obtained in the G1–TC and G3–TC+BG combinations (3.98 ± 0.01 EU/mL), followed by G2–TC+BG (3.36 ± 0.01) and G3–TC+BG+VC (3.21 ± 0.01). In contrast, the lowest CAT activities were recorded in G2–TC+VC (0.61 ± 0.01), G2–TC+CM, and G2–TC (each 0.92 ± 0.01) combinations (Figure 3A).

Ascorbate peroxidase (APX) activity was also significantly influenced by both genotype and treatment (*p* ≤ 0.001). The highest APX activity was measured in genotype G3 (0.15 ± 0.01 EU/mL), followed by G2 (0.07 ± 0.01) and G1 (0.05 ± 0.01). Genotype G1 exhibited significantly lower APX activity compared to the others. Among the treatments, the highest activity was recorded in TC+BG+VC (0.19 ± 0.01 EU/mL), whereas the lowest was found in TC+CM+BG (0.05 ± 0.00) (Table 2).

The genotype × treatment interaction also had a significant effect on APX activity (*p* ≤ 0.001). The G3–TC+BG+VC combination exhibited the highest activity (0.50 ± 0.01 EU/mL), followed by G2–TC+BG (0.18 ± 0.01) and G3–TC+VC (0.12 ± 0.01). The lowest APX activities were recorded in the G2–TC+VC (0.02 ± 0.01) and G1–TC+BG+VC (0.03 ± 0.01) combinations (Figure 3B).

### 5.4 Chlorophyll a Content

Statistically significant differences were observed among maize genotypes in terms of chlorophyll a (Chl a) content (*p* ≤ 0.001). The highest Chl a level was recorded in genotype G3 (1.11 ± 0.01 mg/g FW), followed by G2 (0.96 ± 0.01) and G1 (0.65 ± 0.01). Treatment type also had a significant effect on Chl a content (*p* ≤ 0.001). The TC+BG application resulted in the highest Chl a level (1.41 ± 0.01 mg/g FW), followed by TC (1.01 ± 0.01) and TC+CM (0.99 ± 0.01). The lowest value was observed under the TC+CM+BG treatment (0.43 ± 0.01) (Table 3).

**TABLE 3:** Mean values and significance levels of chlorophyll a and b, total chlorophyll affected by different genotypes and applications.

Genotype		Chl a	Genotype		Chl b	Genotype		TChl
*G3*	a	1.11 ± 0.01	*G3*	a	0.41 ± 0.01	*G3*	a	1.52 ± 0.01
*G2*	b	0.96 ± 0.01	*G2*	b	0.39 ± 0.01	*G2*	b	1.35 ± 0.01
*G1*	c	0.65 ± 0.01	*G1*	c	0.28 ± 0.01	*G1*	c	0.93 ± 0.01
CV (%)		1.08	CV (%)		2.72	CV (%)		0.77
Chl a		***	Chl b		***	TChl		***

Applications		Chl a	Applications		Chl b	Applications		TChl
*TC*+*BG*	a	1.41 ± 0.01	*TC*+*BG*	a	0.50 ± 0.01	*TC*+*BG*	a	1.91 ± 0.01
*TC*	b	1.01 ± 0.01	*TC*	b	0.40 ± 0.01	*TC*	b	1.41 ± 0.01
*TC*+*CM*	c	0.99 ± 0.01	*TC*+*CM*	c	0.38 ± 0.01	*TC*+*CM*	c	1.38 ± 0.01
*TC*+*BG*+*VC*	d	0.82 ± 0.01	*TC*+*BG*+*VC*	d	0.35 ± 0.01	*TC*+*BG*+*VC*	d	1.17 ± 0.01
*TC*+*VC*	e	0.77 ± 0.01	*TC*+*VC*	e	0.33 ± 0.01	*TC*+*VC*	e	1.10 ± 0.01
*TC*+*CM*+*BG*	f	0.43 ± 0.01	*TC*+*CM*+*BG*	f	0.21 ± 0.01	*TC*+*CM*+*BG*	f	0.64 ± 0.01
CV (%)		1.08	CV (%)		2.72	CV (%)		0.77
Chl a		***	Chl b		***	TChl		***

*Note:* *p* ≤ 0.01.

Abbreviations: BG, Bat Guano; Chl a, Chlorophyll a (mg/g FW); Chl b, Chlorophyll b (mg/g FW); CM, Chicken Manure; CV, Coefficient of Variation Value; G1, G2, G3, Genotype 1, Genotype 2, Genotype 3; N.S., not significant; TC, Tea Compost; TChl, Total Chlorophyll (mg/g FW); VC, Vermicompost.

*** *p* ≤ 0.001.

**FIGURE 4:**
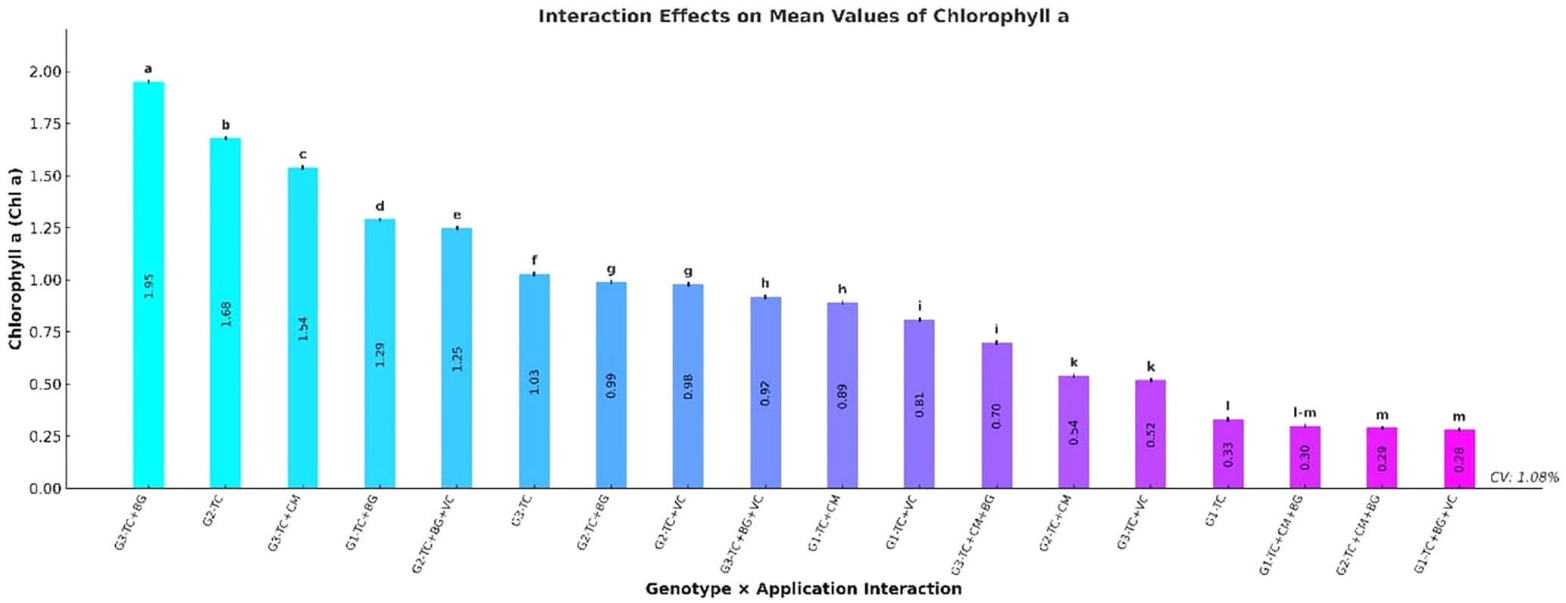
Effects of genotype × treatment interactions on chlorophyll *a* content (Chl *a*) (mg/g FW). Different letters indicate statistically significant differences among means based on Tukey's test (*p* ≤ 0.001). Groups sharing the same letter are not significantly different. BG, Bat guano; CM, Chicken manure; G1, G2, G3, Genotype 1, Genotype 2, Genotype 3; TC, Tea compost; VC, Vermicompost.

The genotype × treatment interaction exhibited a highly significant influence on Chl a levels (*p* ≤ 0.001). The highest value was measured in the G3–TC+BG combination (1.95 ± 0.01 mg/g FW), followed by G2–TC (1.68 ± 0.01) and G3–TC+CM (1.54 ± 0.01). Other notable combinations with elevated Chl a content included G1–TC+BG (1.29 ± 0.01) and G2–TC+BG+VC (1.25 ± 0.01). In contrast, the lowest Chl a levels were found in G2–TC+BG+VC (0.28 ± 0.01) and G2–TC+CM+BG (0.29 ± 0.01) (Figure 4).

### 5.5 Chlorophyll b Content

Chlorophyll b (Chl b) content differed significantly among genotypes (*p* ≤ 0.001). The highest Chl b level was observed in genotype G3 (0.41 ± 0.01 mg/g FW), followed by G2 (0.39 ± 0.01) and G1 (0.28 ± 0.01). With respect to treatments, the TC+BG application yielded the highest Chl b content (0.50 ± 0.01), followed by TC (0.40 ± 0.01) and TC+CM (0.38 ± 0.01). The lowest Chl b level was detected under the TC+CM+BG treatment (0.21 ± 0.01) (Table 3).

**FIGURE 5:**
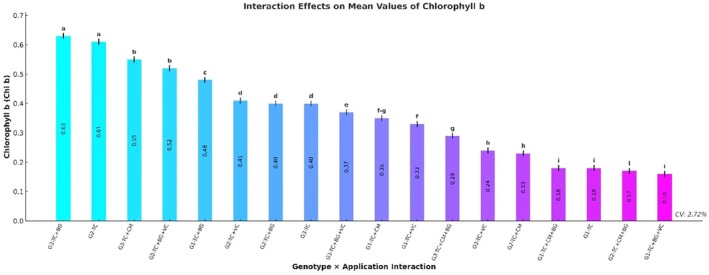
Effects of genotype × treatment interactions on chlorophyll b content (Chl b) (mg/g FW). Different letters indicate statistically significant differences among means based on Tukey's test (*p* ≤ 0.001). Groups sharing the same letter are not significantly different. BG, Bat Guano; CM, Chicken Manure; G1, G2, G3, Genotype 1, Genotype 2, Genotype 3; TC, Tea Compost; VC, Vermicompost.

The genotype × treatment interaction also had a highly significant effect on Chl b content (*p* ≤ 0.001). The G3–TC+BG (0.63 ± 0.01 mg/g FW) and G2–TC (0.61 ± 0.01) combinations exhibited the highest Chl b levels. In contrast, the lowest values were observed in the G1–TC+BG+VC (0.16 ± 0.01) and G2–TC+CM+BG (0.17 ± 0.01) combinations (Figure 5).

### 5.6 Total Chlorophyll Content

Total chlorophyll (TChl) content varied significantly among genotypes (*p* ≤ 0.001). The highest TChl value was recorded in genotype G3 (1.52 ± 0.01 mg/g FW), followed by G2 (1.35 ± 0.01) and G1 (0.93 ± 0.01). In terms of treatments, the TC+BG application resulted in the highest TChl content (1.91 ± 0.01), followed by TC (1.41 ± 0.01) and TC+CM (1.38 ± 0.01). The lowest total chlorophyll level was observed under the TC+CM+BG treatment (0.64 ± 0.01) (Table 3).

**FIGURE 6:**
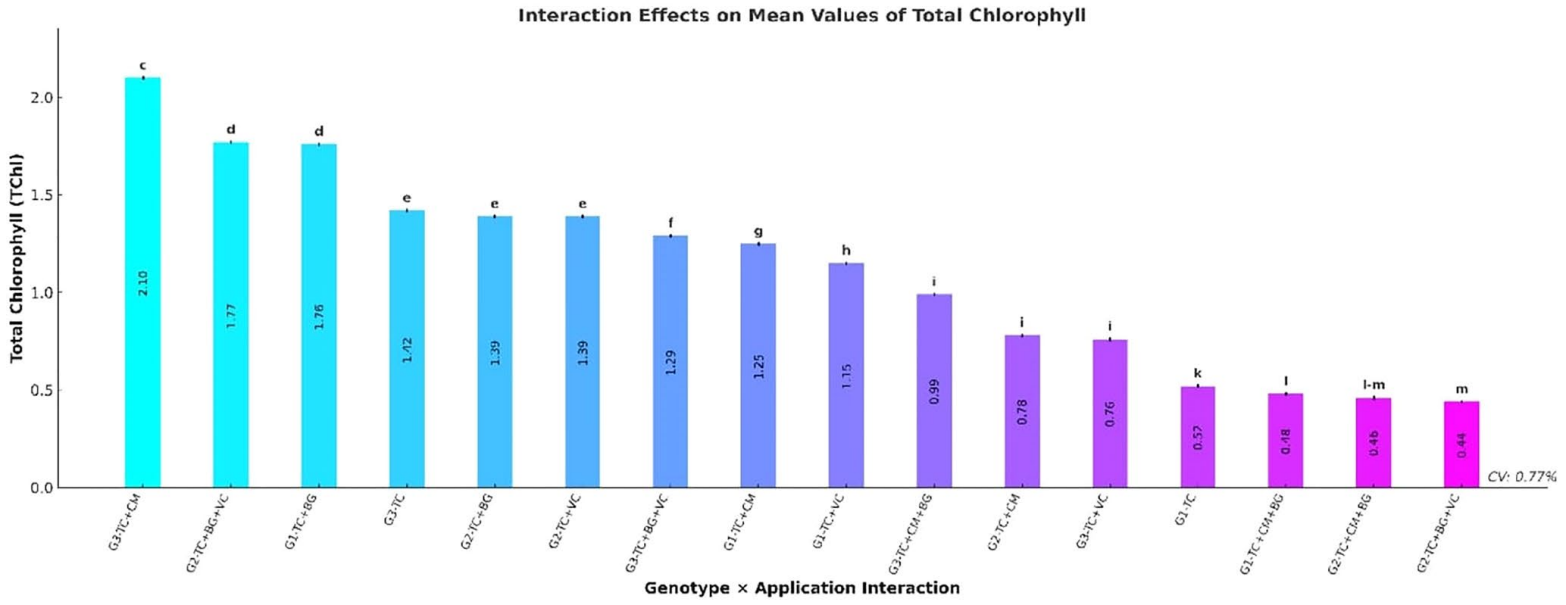
Effects of genotype × treatment interactions on total chlorophyll content (TChl) (mg/g FW). Different letters indicate statistically significant differences among group means according to Tukey's test (*p* ≤ 0.001). Groups sharing the same letter are not significantly different. BG, Bat Guano; CM, Chicken Manure; G1, G2, G3, Genotype 1, Genotype 2, Genotype 3; TC, Tea Compost; VC, Vermicompost.

A highly significant genotype × treatment interaction was also found for TChl levels (*p* ≤ 0.001). The greatest total chlorophyll accumulation was detected in the G3–TC+CM combination (2.10 ± 0.01 mg/g FW), followed by G2–TC+BG+VC (1.77 ± 0.01) and G1–TC+BG (1.76 ± 0.01). Notably, the lowest TChl values were also reported in G2–TC+BG+VC and G3–TC+CM+BG (0.44 and 0.46 ± 0.01, respectively), which fell into the same statistical group (Figure 6).

### 5.7 Chlorophyll a/b Ratio

The chlorophyll a/b (Chl a/b) ratio varied significantly among genotypes (*p* ≤ 0.001). The highest Chl a/b ratio was recorded in genotype G3 (2.58 ± 0.01), followed by G2 (2.35 ± 0.01) and G1 (2.17 ± 0.01). In terms of treatments, the TC+BG application resulted in the highest ratio (2.76 ± 0.01), followed by TC+CM (2.55 ± 0.01) and TC (2.39 ± 0.01). The lowest Chl a/b ratio was observed in the TC+CM+BG treatment (1.95 ± 0.01) (Table 4).

**TABLE 4:** Mean values and significance levels of chlorophyll a/b ratio and total carotenoid affected by different genotypes and applications.

Genotype		Chl a/b	Genotype		TC
*G3*	a	2.58 ± 0.01	*G3*	a	0.31 ± 0.01
*G2*	b	2.35 ± 0.01	*G2*	b	0.26 ± 0.01
*G1*	c	2.17 ± 0.01	*G1*	c	0.18 ± 0.01
CV (%)		0.41	CV (%)		3.93
Chl a/b		***	TC		***

Applications		Chl a/b	Applications		TC
*TC*+*BG*	a	2.76 ± 0.01	*TC*+*BG*	a	0.38 ± 0.01
*TC*+*CM*	b	2.55 ± 0.01	*TC*+*CM*	b	0.28 ± 0.01
*TC*	c	2.39 ± 0.01	*TC*	b	0.28 ± 0.01
*TC*+*VC*	d	2.34 ± 0.01	*TC*+*BG*+*VC*	c	0.23 ± 0.01
*TC*+*BG*+*VC*	e	2.21 ± 0.01	*TC*+*VC*	d	0.20 ± 0.01
*TC*+*CM*+*BG*	f	1.95 ± 0.01	*TC*+*CM*+*BG*	e	0.12 ± 0.01
CV (%)		0.41	CV (%)		3.93
Chl a/b		***	TC		***

Abbreviations: BG, Bat Guano; Chl a/b, Chlorophyll a/b Ratio; CM, Chicken Manure; CV, Coefficient of Variation Value; G1, G2, G3, Genotype 1, Genotype 2, Genotype 3; N.S., not significant; TC, Tea Compost; TC, Total Carotenoid (mg/g FW); VC, Vermicompost.

*** *p* ≤ 0.001.

**FIGURE 7:**
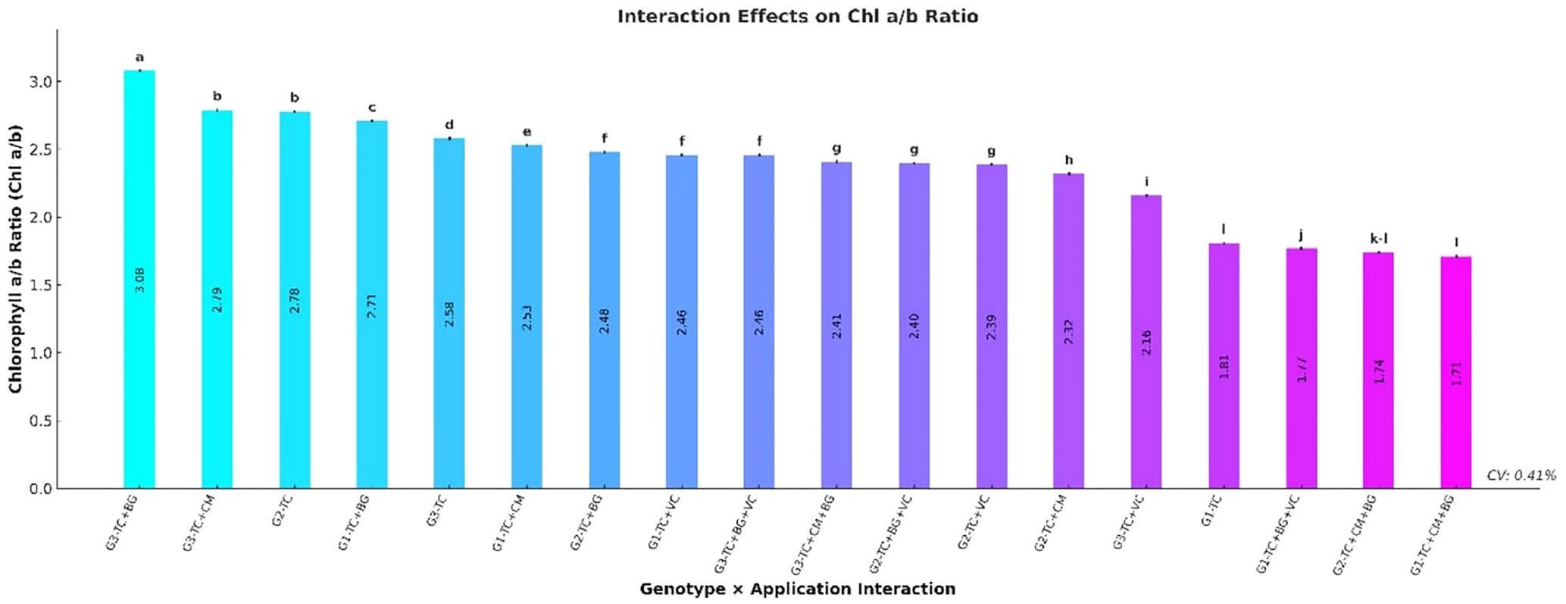
Effects of genotype × treatment interactions on chlorophyll a/b ratio (Chl a/b). Different letters indicate statistically significant differences among group means according to Tukey's test (*p* ≤ 0.001). Groups sharing the same letter are not significantly different. BG, Bat Guano; CM, Chicken Manure; G1, G2, G3, Genotype 1, Genotype 2, Genotype 3; TC, Tea Compost; VC, Vermicompost.

The genotype × treatment interaction also had a significant effect on the Chl a/b ratio (*p* ≤ 0.001). The highest ratio was measured in the G3–TC+BG combination (3.08 ± 0.01), followed by G3–TC+CM (2.79 ± 0.01) and G2–TC (2.78 ± 0.01). Other notable high ratios were recorded in G1–TC+BG (2.71 ± 0.01) and G3–TC (2.58 ± 0.01). In contrast, the lowest Chl a/b ratios were observed in the G1–TC+CM+BG (1.71 ± 0.01) and G2–TC+CM+BG (1.74 ± 0.01) combinations (Figure 7).

### 5.8 Total Carotenoid Content

Total carotenoid content differed significantly among genotypes (*p* ≤ 0.001). The highest carotenoid level was recorded in genotype G3 (0.31 ± 0.01 mg/g FW), followed by G2 (0.26 ± 0.01) and G1 (0.18 ± 0.01). In terms of treatments, the TC+BG application resulted in the highest carotenoid content (0.38 ± 0.01 mg/g FW), followed by TC+CM and TC (both 0.28 ± 0.01). The lowest carotenoid concentration was observed in the TC+CM+BG treatment (0.12 ± 0.01) (Table 4).

**FIGURE 8:**
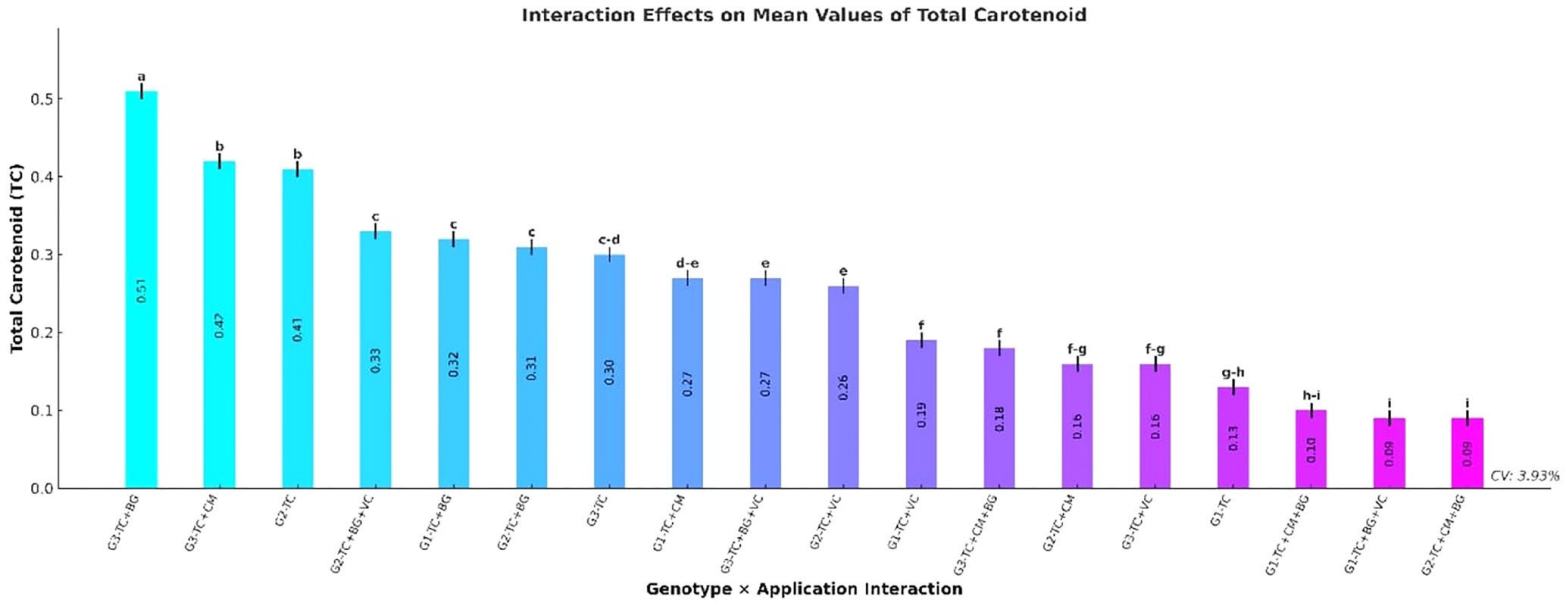
Effects of genotype × treatment interactions on total carotenoid content (TC) (mg/g FW). Different letters indicate statistically significant differences among group means according to Tukey's test (*p* ≤ 0.001). Groups sharing the same letter are not significantly different. BG, Bat Guano; CM, Chicken Manure; G1, G2, G3, Genotype 1, Genotype 2, Genotype 3; TC, Tea Compost; VC, Vermicompost.

The genotype × treatment interaction also had a statistically significant effect on total carotenoid content (*p* ≤ 0.001). The highest value was observed in the G3–TC+BG combination (0.51 ± 0.01 mg/g FW), followed by G3–TC+CM (0.42 ± 0.01) and G2–TC (0.41 ± 0.01). Moderate levels of carotenoid accumulation were recorded in G2–TC+BG+VC (0.33 ± 0.01) and G3–TC (0.30 ± 0.01). The lowest carotenoid concentrations were found in G2–TC+CM+BG and G1–TC+BG+VC, both measuring 0.09 ± 0.01 mg/g FW (Figure 8).

### 5.9 Plant Stem Length, Number of Leaves, and Leaf Length

Stem length differed significantly among genotypes (*p* ≤ 0.001). The greatest stem length was recorded in genotype G1 (77.68 ± 3.01 cm), followed by G2 (62.15 ± 3.01) and G3 (57.90 ± 3.01). Treatment types also had a significant effect on stem length (*p* ≤ 0.05). The longest plants were observed under the TC+CM application (71.11 ± 4.25 cm), followed by TC+CM+BG (70.61 ± 4.25) and TC+BG (70.03 ± 4.25). The shortest plants were recorded in the TC treatment group (58.56 ± 4.25) (Table 5).

**TABLE 5:** Mean values and significance levels of plant stem length, number of leaves, and leaf length affected by different genotypes and applications.

Genotype		PSL	Genotype		NL	Genotype		LL
*G1*	a	77.68 ± 3.01	*G1*	—	7.33 ± 0.25	*G3*	a	53.66 ± 2.86
*G2*	b	62.15 ± 3.01	*G2*	—	7.17 ± 0.25	*G1*	a	52.33 ± 2.86
*G3*	b	57.90 ± 3.01	*G3*	—	6.75 ± 0.25	*G2*	b	38.60 ± 2.86
CV (%)		13.68	CV (%)		10.48	CV (%)		17.79
PSL		***	NL		N.S.	LL		***

Applications		PSL	Applications		NL	Applications		LL
*TC*+*CM*	a	71.11 ± 4.25	*TC*+*CM*	a	8.00 ± 0.35	*TC*+*CM*+*BG*	—	53.77 ± 4.04
*TC*+*CM*+*BG*	ab	70.61 ± 4.25	*TC*+*BG*	ab	7.22 ± 0.35	*TC*	—	50.41 ± 4.04
*TC*+*BG*	ab	70.03 ± 4.25	*TC*+*VC*	ab	7.06 ± 0.35	*TC*+*CM*	—	48.28 ± 4.04
*TC*+*VC*	ab	64.23 ± 4.25	*TC*+*BG*+*VC*	b	6.89 ± 0.35	*TC*+*BG*+*VC*	—	48.28 ± 4.04
*TC*+*BG*+*VC*	ab	60.33 ± 4.25	*TC*	b	6.67 ± 0.35	*TC*+*BG*	—	46.61 ± 4.04
*TC*	b	58.56 ± 4.25	*TC*+*CM*+*BG*	b	6.67 ± 0.35	*TC*+*VC*	—	44.23 ± 4.04
CV (%)		13.68	CV (%)		10.48	CV (%)		17.79
PSL		**	NL		***	LL		N.S.

Abbreviations: BG, Bat Guano; CM, Chicken Manure; CV, Coefficient of Variation Value; G1, G2, G3, Genotype 1, Genotype 2, Genotype 3; N.S., not significant; NL, Number of Leaves; PSL, Plant Stem Length (cm); TC, Tea Compost; VC, Vermicompost.

** *p* ≤ 0.01.

*** *p* ≤ 0.001.

A highly significant genotype × treatment interaction was also detected for stem length (*p* ≤ 0.001). The longest plants were observed in the G1–TC+VC (86.07 ± 7.37 cm) and G1–TC+CM+BG (85.33 ± 7.37) combinations. Other notable high values were recorded in G1–TC+BG (81.77 ± 7.37) and G1–TC+BG+VC (81.00 ± 7.37). The shortest plants were observed in the G3–TC (37.20 ± 7.37) and G2–TC+BG+VC (43.00 ± 7.37) combinations (Table 6).

**TABLE 6:** Interaction effects (genotype × application) on mean values and significance levels of plant stem length, number of leaves, leaf length.

Interaction		PSL	Interaction		NL	Interaction		LL
*G1−TC*+*VC*	a	86.07 ± 7.37	*G1−TC*+*CM*	—	8.67 ± 0.61	*G3−TC*	—	64.20 ± 7.00
*G1−TC*+*CM*+*BG*	a	85.33 ± 7.37	*G3−TC*	—	8.67 ± 0.61	*G1−TC*+*CM*+*BG*	—	63.17 ± 7.00
*G1−TC*+*BG*	ab	81.77 ± 7.37	*G3−TC*+*CM*	—	7.67 ± 0.61	*G1−TC*+*CM*+*VC*	—	57.13 ± 7.00
*G1−TC*+*BG*+*VC*	ab	81.00 ± 7.37	*G1−TC*+*VC*	—	7.67 ± 0.61	*G1−TC*+*BG*+*VC*	—	56.80 ± 7.00
*G2−TC*+*CM*	ab	79.73 ± 7.37	*G2−TC*+*CM*	—	7.67 ± 0.61	*G1−TC*+*CM*	—	53.67 ± 7.00
*G2−TC*	a–c	76.13 ± 7.37	*G2−TC*+*BG*	—	7.33 ± 0.61	*G1−TC*+*VC*	—	51.70 ± 7.00
*G3−TC*+*BG*	a–c	72.60 ± 7.37	*G1−TC*+*BG*	—	7.33 ± 0.61	*G3−TC*+*CM*	—	51.43 ± 7.00
*G1−TC*+*CM*	a–d	69.00 ± 7.37	*G2−TC*	—	7.33 ± 0.61	*G1−TC*+*BG*	—	50.17 ± 7.00
*G2−TC*+*CM*+*BG*	a–d	67.00 ± 7.37	*G3−TC*+*BG*	—	7.00 ± 0.61	*G3−TC*+*BG*+*VC*	—	49.23 ± 7.00
*G3−TC*+*CM*	a–d	66.40 ± 7.37	*G2−TC*+*VC*	—	7.00 ± 0.61	*G1−TC*	—	49.10 ± 7.00
*G1−TC*	a–e	62.33 ± 7.37	*G3−TC*+*BG*+*VC*	—	7.00 ± 0.61	*G1−TC*+*BG*	—	48.23 ± 7.00
*G3−TC*+*CM*+*BG*	a–e	59.30 ± 7.37	*G2−TC*+*BG*+*VC*	—	7.00 ± 0.61	*G3−TC*+*VC*	—	41.10 ± 7.00
*G3−TC*+*BG*+*VC*	b–e	56.40 ± 7.37	*G1−TC*+*CM*+*BG*	—	6.67 ± 0.61	*G2−TC*+*CM*+*BG*	—	41.00 ± 7.00
*G2−TC*+*BG*	b–e	55.10 ± 7.37	*G2−TC*+*CM*+*BG*	—	6.67 ± 0.61	*G2−TC*+*VC*	—	39.90 ± 7.00
*G3−TC*+*VC*	b–e	55.00 ± 7.37	*G1−TC*	—	6.67 ± 0.61	*G2−TC*+*CM*	—	39.73 ± 7.00
*G2−TC*+*VC*	c–e	51.13 ± 7.37	*G1−TC*+*BG*+*VC*	—	6.50 ± 0.61	*G2−TC*	—	37.93 ± 7.00
*G2−TC*+*BG*+*VC*	d–e	43.00 ± 7.37	*G3−TC*+*VC*	—	6.33 ± 0.61	*G2−TC*+*BG*+*VC*	—	37.60 ± 7.00
*G3−TC*	e	37.20 ± 7.37	*G3−TC*+*CM*+*BG*	—	6.00 ± 0.61	*G2−TC*+*BG*	—	35.43 ± 7.00
CV (%)		13.68	CV (%)		10.48	CV (%)		17.79
PSL		***	NL		N.S.	LL		N.S.

Abbreviations: BG, Bat Guano; CM, Chicken Manure; CV, Coefficient of Variation Value; G1, G2, G3, Genotype 1, Genotype 2, Genotype 3; LL, Leaf Length (cm); N.S., not significant; NL, Number of Leaves; PSL, Plant Stem Length (cm); TC, Tea Compost; VC, Vermicompost.

*** *p* ≤ 0.001.

No statistically significant differences were found among genotypes in terms of leaf number. Average leaf counts were relatively similar: G1 (7.33), G2 (7.17), and G3 (6.75) (Table 5).

In contrast, treatment types significantly affected leaf number (*p* ≤ 0.001). The highest leaf count was observed in the TC+CM application (8.00 ± 0.35), followed by TC+BG (7.22 ± 0.35) and TC+VC (7.06 ± 0.35). The lowest leaf counts were recorded in TC+CM+BG and TC treatments (both 6.67 ± 0.35) (Table 5).

Although genotype × treatment interaction did not have a statistically significant effect on leaf number, several notable trends were observed. The highest leaf numbers were found in G1–TC+CM and G3–TC combinations (both 8.67 ± 0.61). Other high values were recorded in G3–TC+CM and G1–TC+VC (7.67 ± 0.61). The lowest leaf counts were observed in G2–TC+CM+BG (6.00 ± 0.61) and G2–TC+VC (6.33 ± 0.61) (Table 6).

Leaf length was significantly influenced by genotype (*p* ≤ 0.001). The longest leaves were recorded in genotype G3 (53.66 ± 2.86 mm), followed by G1 (52.33 ± 2.86) and G2 (38.60 ± 2.86). Both G3 and G1 showed significantly longer leaves compared to G2 (Table 5).

No statistically significant differences were found among treatments in terms of leaf length. However, based on mean values, the longest leaves were recorded in the TC+CM+BG treatment (53.77 ± 4.04 mm), while the shortest were found in TC+VC (44.23 ± 4.04) and TC+BG (46.61 ± 4.04) treatments (Table 5).

Although the genotype × treatment interaction did not have a statistically significant effect on leaf length, notable variations were observed in the mean values. The longest leaves were recorded in the G3–TC (64.20 ± 7.00 mm) and G1–TC+CM+BG (63.17 ± 7.00) combinations, while the shortest were observed in G2–TC+BG (35.43 ± 7.00) and G2–TC+BG+VC (37.60 ± 7.00) combinations (Table 6).

### 5.10 Leaf Width, Leaf Area, and Fresh Seedling Weight

Leaf width did not differ significantly among genotypes. Based on mean values, the highest leaf width was recorded in genotype G3 (4.48 ± 0.30 cm), followed by G1 (3.97 ± 0.30) and G2 (3.83 ± 0.30) (Table 7).

**TABLE 7:** Mean values and significance levels of leaf width, leaf area, and fresh seedling weight affected by different genotypes and applications.

Genotype		LW	Genotype		LA	Genotype		FSW
*G3*	—	4.48 ± 0.30	*G3*	a	181.82 ± 14.85	*G1*	—	65.00 ± 9.33
*G1*	—	3.97 ± 0.30	*G1*	a	155.31 ± 14.85	*G3*	—	61.25 ± 9.33
*G2*	—	3.83 ± 0.30	*G2*	b	110.50 ± 14.85	*G2*	—	43.61 ± 9.33
CV (%)		21.94	CV (%)		29.87	CV (%)		49.43
LW		N.S.	LA		***	FSW		N.S.

Applications		LW	Applications		LA	Applications		FSW
*TC*+*CM*	—	4.60 ± 0.42	*TC*+*CM*	—	169.19 ± 21.01	*TC*+*CM*	a	80.56 ± 13.19
*TC*+*VC*	—	4.34 ± 0.42	*TC*+*BG*+*VC*	—	155.75 ± 21.01	*TC*+*BG*	ab	62.78 ± 13.19
*TC*+*BG*+*VC*	—	4.22 ± 0.42	*TC*+*CM+BG*	—	152.07 ± 21.01	*TC+VC*	ab	60.28 ± 13.19
*TC+BG*	—	4.00 ± 0.42	*TC+VC*	—	142.94 ± 21.01	*TC+BG+VC*	ab	50.00 ± 13.19
*TC+CM+BG*	—	3.76 ± 0.42	*TC*	—	140.60 ± 21.01	*TC+CM+BG*	ab	48.89 ± 13.19
*TC*	—	3.64 ± 0.42	*TC+BG*	—	134.69 ± 21.01	*TC*	b	37.22 ± 13.19
CV (%)		21.94	CV (%)		29.87	CV (%)		49.43
LW		N.S.	LA		N.S.	FSW		**

Abbreviations: BG, Bat Guano; CM, Chicken Manure; CV, Coefficient of Variation Value; FSW, Fresh Seedling Weight (g); G1, G2, G3, enotype 1, Genotype 2, Genotype 3; LA, Leaf Area (cm^2^); LL, Leaf Length (cm); LW, Leaf Width (cm); N.S., not significant; TC, Tea Compost; VC, Vermicompost.

** *p* ≤ 0.01.

*** *p* ≤ 0.001.

Similarly, no statistically significant differences were found among treatments regarding leaf width. According to mean values, the greatest leaf width was observed under the TC+CM treatment (4.60 ± 0.42 cm), followed by TC+VC (4.34 ± 0.42) and TC+BG+VC (4.22 ± 0.42). The lowest value was recorded under the TC treatment (3.64 ± 0.42) (Table 7).

The genotype × treatment interaction had no statistically significant effect on leaf width. Nevertheless, based on mean values, the highest LW was recorded in the G3–TC+CM combination (5.17 ± 0.73 cm), followed by G1–TC+CM (4.67 ± 0.73) and both G3–TC+CM+BG and G3–TC+VC (4.53 ± 0.73). The lowest values were measured in G1–TC (3.87 ± 0.73) and G2–TC+CM+BG (3.93 ± 0.73) (Table 8).

**TABLE 8:** Interaction effects (genotype × application) on mean values and significance levels of leaf width, leaf area, and fresh seedling weight.

Interaction		LW	Interaction		LA	Interaction		FSW
*G3−TC+CM*	—	5.17 ± 0.73	*G3−TC*	—	202.23 ± 36.38	G3−TC+CM	—	96.67 ± 22.85
*G1−TC+CM*	—	4.67 ± 0.73	*G3−TC+CM*	—	199.98 ± 36.38	G1−TC+CM	—	95.00 ± 22.85
*G3−TC+CM+BG*	—	4.53 ± 0.73	*G3−TC+CM+BG*	—	199.05 ± 36.38	G1−TC+VC	—	78.33 ± 22.85
*G3−TC+VC*	—	4.53 ± 0.73	*G1−TC+CM*	—	185.19 ± 36.38	G3−TC+BG	—	73.33 ± 22.85
*G1−TC+VC*	—	4.47 ± 0.73	*G1−TC+BG+VC*	—	183.51 ± 36.38	G1−TC+BG	—	70.00 ± 22.85
*G3−TC+BG+VC*	—	4.47 ± 0.73	*G3−TC+VC*	—	173.94 ± 36.38	G3−TC+CM+BG	—	65.00 ± 22.85
*G3−TC*	—	4.20 ± 0.73	*G1−TC+CM+BG*	—	166.29 ± 36.38	G1−TC+CM+BG	—	58.33 ± 22.85
*G2−TC+BG+VC*	—	4.20 ± 0.73	*G3−TC+BG+VC*	—	165.53 ± 36.38	G1−TC+BG+VC	—	56.67 ± 22.85
*G1−TC+BG+VC*	—	4.17 ± 0.73	*G1−TC+BG*	—	152.01 ± 36.38	G2−TC+VC	—	50.00 ± 22.85
*G2−TC+VC*	—	4.17 ± 0.73	*G3−TC+BG*	—	150.17 ± 36.38	G2−TC+CM	—	50.00 ± 22.85
*G3−TC+BG*	—	4.17 ± 0.73	*G1−TC+VC*	—	132.59 ± 36.38	G2−TC+BG+VC	—	50.00 ± 22.85
*G2−TC+CM*	—	4.00 ± 0.73	*G2−TC+CM*	—	122.41 ± 36.38	G3−TC+VC	—	47.50 ± 22.85
*G1−TC+BG*	—	4.00 ± 0.73	*G2−TC+VC*	—	122.30 ± 36.38	G2−TC	—	45.00 ± 22.85
*G2−TC+BG*	—	3.97 ± 0.73	*G2−TC+BG+VC*	—	118.21 ± 36.38	G2−TC+BG	—	45.00 ± 22.85
*G2−TC*	—	3.93 ± 0.73	*G1−TC*	—	112.25 ± 36.38	G2−TC+BG+VC	—	43.33 ± 22.85
*G1−TC+CM+BG*	—	3.93 ± 0.73	*G2−TC*	—	107.39 ± 36.38	G3−TC	—	35.00 ± 22.85
*G2−TC+CM+BG*	—	3.93 ± 0.73	*G2−TC+BG*	—	101.90 ± 36.38	G1−TC	—	31.67 ± 22.85
*G1−TC*	—	3.87 ± 0.73	*G2−TC+CM+BG*	—	90.86 ± 36.38	G2−TC+CM+BG	—	23.33 ± 22.85
CV (%)		21.94	CV (%)		29.87	CV (%)		49.43
LW		N.S.	LA		N.S.	FSW		N.S.

Abbreviations: BG, Bat Guano; CM, Chicken Manure; CV, Coefficient of Variation Value; FSW, Fresh Seedling Weight (g); G1, G2, G3, Genotype 1, Genotype 2, Genotype 3; LA, Leaf Area (cm^2^); LW, Leaf Width (cm); N.S., not significant; TC, Tea Compost; VC, Vermicompost.

Leaf area (LA) varied significantly among genotypes (*p* ≤ 0.001). The largest leaf area was recorded in genotype G3 (181.82 ± 14.85 cm^2^), followed by G1 (155.31 ± 14.85) and G2 (110.50 ± 14.85) (Table 7).

However, no statistically significant differences were found among treatments regarding leaf area. According to mean values, the highest LA was observed in the TC+CM treatment (169.19 ± 21.01 cm^2^), followed by TC+BG+VC (155.75 ± 21.01) and TC+CM+BG (152.07 ± 21.01). The lowest leaf area was recorded under the TC+BG treatment (134.69 ± 21.01) (Table 7).

The genotype × treatment interaction for LA was not statistically significant. Nonetheless, the highest mean LA values were recorded in G3–TC (202.23 ± 36.35 cm^2^) and G3–TC+CM (199.98 ± 36.35), followed by G3–TC+CM+BG (199.05 ± 36.35) and G1–TC+CM (185.19 ± 36.35). The lowest LA values were recorded in G2–TC+CM+BG (90.86 ± 36.35) and G2–TC+BG (101.90 ± 36.35) (Table 8).

Fresh seedling weight (FSW) did not differ significantly among genotypes. Based on mean values, genotype G1 had the highest FSW (65.00 ± 9.33 g), followed by G3 (61.25 ± 9.33) and G2 (43.61 ± 9.33) (Table 7).

On the other hand, FSW differed significantly among treatments (*p* ≤ 0.05). The highest FSW value was observed in the TC+CM treatment (80.56 ± 13.19 g), followed by TC+BG (62.78 ± 13.19), TC+VC (60.28 ± 13.19), and TC+BG+VC (50.00 ± 13.19). The lowest FSW was recorded under the TC treatment (37.22 ± 13.19) (Table 7).

Although the genotype × treatment interaction was not statistically significant for FSW, some differences were evident based on mean values. The highest FSW was observed in G3–TC+CM (96.67 ± 22.85 g) and G1–TC+CM (95.00 ± 22.85), followed by G1–TC+VC (78.33 ± 22.85) and G3–TC+BG (73.35 ± 22.85). The lowest fresh seedling weights were measured in G2–TC+CM+BG (23.33 ± 22.85) and G1–TC (31.67 ± 22.85) (Table 8).

## 6 Discussion

Significant effects of genotype and treatment were observed on total phenolic content (TPC) and total flavonoid content (TFC) (*p* ≤ 0.001). While the genotype × treatment interaction was not significant for TPC, it was statistically significant for TFC. Among the genotypes, the highest TPC value was recorded in G2 (194.74 ± 5.85 mg GAE/g DW), followed by G1 (181.98 ± 5.85) and G3 (167.77 ± 5.85). The G2 genotype exhibited the highest total flavonoid content (136.47 ± 0.28 mg QE/g DW). Regarding treatments, the highest TFC value was observed under the TC+BG+VC application (138.83 ± 0.55 mg QE/g DW), followed by TC+BG (134.56 ± 0.55) and TC+VC (129.42 ± 0.55). These findings suggest that the synthesis of plant defense compounds is influenced by both the genetic background and the type of applied organic input. Consistently, Ullah et al. (2025) also reported pronounced genotypic differences in maize responses to nitrogen fertilization. Such variation is primarily attributed to the differential expression of genes and enzymes involved in nutrient uptake, transport, and assimilation. Notably, tissue‐specific isoforms of glutamine synthetase (GS) and glutamate synthase (GOGAT) have been identified as key regulators of nitrogen metabolism efficiency (Li et al. 2017; Bernard and Habash 2009). The genotype‐dependent expression of these enzymes may account for the observed variability in the accumulation of defense‐related compounds.

In terms of treatments, the highest total phenolic content (TPC) was recorded in the TC+CM application (194.77 ± 8.28 mg GAE/g DW), followed by TC+BG+VC (192.17 ± 8.28) and TC+VC (187.82 ± 8.28). The lowest TPC value was observed in the TC treatment alone (158.15 ± 8.28 mg GAE/g DW). These findings indicate that the TC+CM treatment markedly enhanced phenolic compound synthesis in maize seedlings, whereas the TC treatment alone appeared to limit their accumulation. According to El‐Leithy et al. (2017), nitrogen‐based fertilizers can influence both primary and secondary metabolic pathways in plants, thereby affecting the biosynthesis of secondary metabolites such as phenolics and flavonoids. It is generally recognized that nutrient‐deficient plants activate stress responses, which in turn promote the accumulation of such compounds (Iqbal et al. 2023). In this context, the chemical composition of the applied fertilizer, the mode of application, and the genetic characteristics of the plant act synergistically to determine the levels of secondary metabolites.

Although the genotype × treatment interaction was not significant for total phenolic content (TPC), it was found to be statistically significant for total flavonoid content (TFC) (*p* ≤ 0.001). The highest TFC value was recorded in the G2–TC+VC combination (195.78 ± 0.96 mg QE/g DW), which was statistically distinct from all other combinations. The lowest TFC levels were detected in G2–TC (93.67 ± 0.96), G1–TC+VC, G3–TC+VC, and G1–TC+CM+BG treatments, ranging from 94.70 to 93.67 mg QE/g DW. While the TC+VC treatment promoted flavonoid accumulation in the G2 genotype, markedly lower levels were observed in G1 and G3 under the same treatment. This discrepancy is likely attributable to an interaction between the genetic background and the nutrient composition of the applied fertilizers. One of the key factors underlying this interaction is the regulatory role of nitrogen in flavonoid metabolism, which has been well documented in the literature. Flavonoid biosynthesis is known to be highly sensitive to nitrogen availability. Several studies have reported that the abundance of flavonoid subclasses—such as flavanones, flavones, flavonols, flavan‐3‐ols, and proanthocyanidins—is inversely related to nitrogen supply (Strissel et al. 2008; Becker et al. 2015). In this regard, considering the nitrogen contents of the organic materials used in our treatments, chicken manure extract (CM) contains approximately 10% total nitrogen, while bat guano (BG) contains around 2% total organic nitrogen. The low TFC values observed in combinations such as G1–TC+CM+BG, G3–TC+VC, and G1–TC+VC are consistent with the literature suggesting that nitrogen‐rich treatments may suppress flavonoid accumulation. Indeed, numerous studies have shown that low‐nitrogen applications significantly enhance the levels of flavonoids and proanthocyanidins. For example, in strawberry and several other species, low nitrogen supply has been reported to increase flavonoid accumulation—particularly in the flavan‐3‐ol and flavonol subclasses (Narvekar and Tharayil 2021). However, the reduced flavonoid content observed under low nitrogen conditions in the G2 genotype does not fully align with the literature that associates nitrogen limitation with increased flavonoid synthesis. This may be attributed to the G2 genotype's limited capacity to activate flavonoid biosynthesis under conditions of extreme nitrogen deficiency.

In our study, total antioxidant capacity (TAC) was significantly affected by both genotype and treatment (*p* ≤ 0.001). The highest TAC value among genotypes was recorded in G3 (95.80 ± 0.07 mg TE/g DW), while the highest value among treatments was observed in the TC+VC application (94.76 ± 0.13 mg TE/g DW), followed by TC+BG (93.67 ± 0.13) and TC (93.06 ± 0.13). The genotype × treatment interaction was also statistically significant for TAC (*p* ≤ 0.001). The highest TAC was detected in the G2–TC+CM+BG combination (97.20 ± 0.23 mg TE/g DW), followed by G3–TC+BG (96.95) and G3–TC+CM (96.80). Notably, the TC+CM+BG treatment applied to G2 resulted in a remarkable increase in TAC, while the TC+BG and TC+CM applications also led to high TAC levels in the G3 genotype. These findings suggest that antioxidant capacity is not only dependent on the composition of the organic materials applied, but also on the genetic background of the plant. According to the literature, the influence of nitrogen‐based fertilization on antioxidant activity may vary depending on both genotype and application dosage. Verma and Shukla (2015) reported that nitrogen can differentially modulate flavonoid accumulation and associated antioxidant activity based on genotype. Similarly, Xu et al. (2021) identified a linear relationship between nitrogen dosage and DPPH scavenging activity in brown rice. Stewart et al. (2000) also demonstrated a direct link between nitrogen levels and flavonoid biosynthesis, which influences the plant's antioxidant defense. In line with these reports, our results indicate that antioxidant capacity varies according to both genotype and nitrogen content and is largely consistent with existing findings. However, the observation that high and low TAC levels varied across different genotypes and treatments suggests that this relationship is not fixed but is rather dynamic and multifactorial, governed by genotype × treatment interactions.

In terms of proline content, only the genotype factor was found to be statistically non‐significant (*p* > 0.05). Nevertheless, numerically higher proline accumulation was observed in the G2 genotype. The effect of treatment was statistically significant (*p* ≤ 0.05), with the highest proline levels recorded under TC+VC (11.13 ± 0.78 μg/μL) and TC+CM+BG (10.69 ± 0.78) applications. A highly significant genotype × treatment interaction was also detected for proline content (*p* ≤ 0.001). The highest proline concentrations were measured in the G3–TC+VC (12.84 ± 1.35 μg/μL), G2–TC+CM+BG (12.10), and G3–TC+BG (11.40) combinations, while the lowest was found in G3–TC+CM (4.58 ± 1.35 μg/μL). These findings indicate that organic materials such as tea compost, chicken manure, bat guano, and vermicompost interact with genotype‐specific metabolic pathways to activate proline accumulation to varying degrees. In particular, TC+VC and TC+CM+BG treatments were found to enhance proline levels. Proline plays a critical role in plant defense against abiotic stresses such as drought by maintaining cellular osmotic balance and supporting stress tolerance (Mahmood et al. 2020; Jie et al. 2020). The elevated proline levels observed under organic treatments likely reflect the activation of these physiological defense mechanisms (Chinsamy et al. 2014; Salehi et al. 2016; Tan et al. 2008). Overall, our results suggest that vermicompost (VC) and the combination of chicken manure and bat guano (CM+BG) hold considerable potential to enhance proline accumulation, thereby contributing to the strengthening of plant stress defense systems. These findings are in agreement with previous reports in the literature.

In our study, catalase (CAT) activity was significantly influenced by both genotype and treatment (*p* ≤ 0.001). The highest CAT activity was observed in genotype G3 (2.37 ± 0.01 EU/mL). Among the treatments, TC+BG yielded the highest CAT activity (3.47 ± 0.01 EU/mL), while the lowest value was recorded under the TC+CM+BG combination (1.07 ± 0.01 EU/mL). The genotype × treatment interaction was also found to be statistically significant (*p* ≤ 0.001). The highest CAT activities were detected in the G1–TC and G3–TC+BG combinations (3.98 ± 0.01 EU/mL), followed by G2–TC+BG (3.36) and G3–TC+BG+VC (3.21). In contrast, the lowest activities were found in G2–TC+VC (0.61), G2–TC+CM, and G2–TC (each 0.92). These findings indicate that, in addition to genetic variation, the composition of the applied organic materials plays a significant role in modulating enzymatic antioxidant responses. The results are also supported by existing literature. For instance, Xie et al. (2024) reported that the combined use of organic and inorganic fertilizers with different soil management practices increased CAT activity in maize by 36.79% to 103.22%. Similarly, Skwaryło‐Bednarz and Krzepiłko (2013) noted that CAT activity is significantly influenced by plant developmental stage, macronutrient supply, and genotype. The high CAT activity observed particularly under the TC+BG treatment in our study is consistent with these previously reported findings.

Ascorbate peroxidase (APX) activity was also significantly affected by both genotype and treatment (*p* ≤ 0.001). Genotype G3 exhibited the highest APX activity (0.15 ± 0.01 EU/mL). Among treatments, the TC+BG+VC combination produced the highest activity (0.19 ± 0.01 EU/mL), while the lowest was observed under TC+CM+BG (0.05 ± 0.00 EU/mL). The genotype × treatment interaction was likewise statistically significant for APX (*p* ≤ 0.001). The G3–TC+BG+VC combination showed the highest activity (0.50 ± 0.01 EU/mL), followed by G2–TC+BG (0.18) and G3–TC+VC (0.12). In contrast, the lowest activities were recorded in G2–TC+VC (0.02) and G1–TC+BG+VC (0.03). These findings highlight the marked impact of both genotype and treatment on APX activity, with the G3 genotype and TC+BG+VC treatment emerging as the most effective combination. Kaur et al. (2022) and Fatma et al. (2016) demonstrated that the co‐application of nitric oxide (NO) and vermicompost (VC) significantly enhances APX activity, contributing to the detoxification of reactive oxygen species (ROS). Tripathi, Mishra, et al. (2017) also reported positive effects of NO and/or VC on antioxidant enzyme activities, with the greatest increases observed when both were applied together. Similarly, El‐Dakak et al. (2021) found that VC enhanced APX activity in broad bean plants under salt stress, supporting improved stress tolerance, while Tripathi, Singh, et al. (2017) noted that SNP application mitigated the adverse effects of silver nanoparticles by increasing APX activity. The elevated APX activity observed under TC+BG+VC in our study is broadly consistent with these literature findings.

In our study, maize genotypes, treatment types, and their interactions significantly influenced both chlorophyll a (Chl a) and chlorophyll b (Chl b) contents (*p* ≤ 0.001). The highest Chl a concentration was observed in genotype G3 and under the TC+BG treatment (1.11 ± 0.01 and 1.41 ± 0.01 mg/g FW, respectively), with the maximum Chl a level recorded in the G3–TC+BG combination (1.95 ± 0.01 mg/g FW). This was followed by G2–TC (1.68 ± 0.01) and G3–TC+CM (1.54 ± 0.01). For Chl b, G3 and the TC+BG treatment again stood out (0.41 mg/g FW), with the highest values found in the G3–TC+BG (0.63 ± 0.01 mg/g FW) and G2–TC (0.61 ± 0.01) combinations. The lowest Chl b concentrations were detected in G1–TC+BG+VC (0.16 ± 0.01) and G2–TC+CM+BG (0.17 ± 0.01). Overall, the highest pigment concentrations for both Chl a and Chl b were generally observed in G3 when combined with the TC+BG treatment, particularly in the G3–TC+BG group, which recorded 1.95 ± 0.01 mg/g FW for Chl a and 0.63 ± 0.01 mg/g FW for Chl b.

Total chlorophyll (TChl) content also showed significant variation across genotypes and treatments (*p* ≤ 0.001). Genotype G3 exhibited the highest TChl concentration (1.52 ± 0.01 mg/g FW), followed by G2 (1.35) and G1 (0.93). Among treatments, TC+BG resulted in the highest total chlorophyll accumulation (1.91 ± 0.01 mg/g FW), followed by TC (1.41) and TC+CM (1.38), whereas TC+CM+BG significantly reduced TChl content (0.64 ± 0.01). The genotype × treatment interaction was also significant for TChl (*p* ≤ 0.001), with the highest value recorded under the G3–TC+CM combination (2.10 ± 0.01 mg/g FW), followed by G2–TC+BG+VC (1.77) and G1–TC+BG (1.76).

As noted in the literature, chlorophyll biosynthesis and stability are directly dependent on the availability of key nutrients such as nitrogen, magnesium, and iron (Karaman et al. 2000). Magnesium, in particular, forms the central atom of the chlorophyll molecule and is thus essential for its structural integrity. A deficiency in Mg can inhibit chlorophyll production and disrupt plant development (Yağmur et al. 2021). Overall, our findings demonstrate that chlorophyll accumulation is not solely influenced by nutrient availability, but also by the genotype of the plant and the composition and interaction of the applied organic materials. Hence, optimizing pigment accumulation may require a holistic nutrient management strategy that takes genotype–treatment compatibility into account. Nitrogen plays a pivotal role in chlorophyll biosynthesis by contributing to protein formation and the structure of photosynthetic enzymes. Adequate nitrogen supply enhances leaf chlorophyll concentration, thereby increasing photosynthetic efficiency and promoting biomass production (Liu et al. 2023). Therefore, assessing chlorophyll levels alongside fresh seedling weight offers a more comprehensive understanding of how photosynthetic capacity influences biomass accumulation.

In our study, although genotype had no significant effect on fresh seedling weight, treatment type did (*p* ≤ 0.05). The highest value was recorded under TC+CM (80.56 ± 13.19 g), while the lowest was found in the TC treatment (37.22 ± 13.19 g). The combinations G3–TC+CM (96.67 ± 22.85 g) and G1–TC+CM (95.00 ± 22.85 g) produced the greatest biomass. When the chlorophyll data are evaluated alongside these findings, it becomes evident that the TC+CM treatment significantly enhanced chlorophyll accumulation and, consequently, photosynthetic capacity. This enhancement is further substantiated by the high fresh biomass values observed in G3 and G1 genotypes, which reached 96.67 g and 95.00 g, respectively.

Total carotenoid content was significantly affected by both genotype and treatment (*p* ≤ 0.001). The highest value was recorded in genotype G3 (0.31 mg/g), while the lowest was observed in G1 (0.18 mg/g). Among treatments, the TC+BG application resulted in the highest carotenoid level (0.38 mg/g), followed by TC+CM and TC (each 0.28 mg/g). The lowest carotenoid concentration was found under the TC+CM+BG treatment (0.12 mg/g). The genotype × treatment interaction was also statistically significant (*p* ≤ 0.001). The highest carotenoid accumulation occurred in the G3–TC+BG (0.51 mg/g) and G3–TC+CM (0.42 mg/g) combinations. Moderate accumulation was noted in G2–TC+BG+VC (0.33 mg/g) and G3–TC (0.30 mg/g). In contrast, the lowest levels were observed in the G2–TC+CM+BG and G1–TC+BG+VC combinations (0.09 mg/g). The notably high carotenoid level in the G3–TC+BG treatment highlights the positive role of this nutrient‐rich organic amendment in supporting carotenoid biosynthesis. These findings align with those of Alahresani and Ramazani (2021), who reported significant effects of both animal‐ and chemical‐based fertilizers on total carotenoid content. On the other hand, the low carotenoid levels observed in treatments such as TC+CM+BG suggest that certain combinations may exert antagonistic effects on carotenoid metabolism.

In our study, plant height, number of leaves, leaf length, leaf width, and leaf area in maize were differentially affected by genotype, treatment, and their interactions. Genotype G1 showed a clear advantage in plant height, while the TC+CM and TC+VC treatments had overall positive effects on morphological traits. Leaf number was significantly affected by treatment, with the highest value recorded under TC+CM. For leaf length and area, genotype G3 performed best, particularly when combined with TC+CM+BG or TC+CM. The literature clearly emphasizes the direct influence of fertilization on the physiological characteristics of maize leaves. For instance, Tian et al. (2021) demonstrated that nitrogen applications can improve leaf area index even under stress conditions such as waterlogging. Similarly, Hafez et al. (2021) reported that the application of spent grain (SG) and *Azospirillum brasilense* significantly enhanced leaf development. These studies reinforce the notion that organic fertilizer applications can have pronounced effects on plant morphology, which are further modulated by genotype–treatment interactions. In particular, treatments with higher nitrogen content appear to support leaf‐related morphological traits, although the magnitude of this effect is likely to depend on genotypic predisposition.

## 7 Conclusions

This study evaluated the effects of tea waste compost enriched with different organic materials on biochemical defense mechanisms, antioxidant capacity, photosynthetic pigment content, and morphological development in maize seedlings across three distinct genotypes. The findings demonstrated that the applied organic mixtures significantly influenced antioxidant defense systems and pigment accumulation in a genotype‐dependent manner. In particular, the TC+CM, TC+VC, and TC+BG treatments stood out by enhancing stress tolerance and promoting seedling growth across multiple parameters. In this context, the valorization of tea waste—a locally available agricultural by‐product—through enrichment with organic inputs not only improved plant performance but also offered a sustainable alternative for environmentally friendly agricultural practices. The results highlight that adopting an integrated approach that considers the interaction between organic inputs and plant genotypes may provide promising opportunities for future environmentally sustainable crop production.

## Author Contributions

G.H.Y.: conceptualization, methodology, software, resources, writing – original draft preparation, writing – review and editing, visualization, supervision, project administration, validation, data curation, funding acquisition, investigation; E.B.A.: biochemical analyses, validation, data curation, funding acquisition, formal analysis; M.D.Ş.: investigation. All authors have read and agreed to the published version of the manuscript.

## Disclosure

The statements, opinions, and data contained in all publications are solely those of the individual author(s) and contributor(s) and not of MDPI and/or the editor(s). MDPI and/or the editor(s) disclaim responsibility for any injury to people or property resulting from any ideas, methods, instructions, or products referred to in the content. This study was supported at all stages under the TÜBİTAK 2209‐A Program by the Scientist Support Programs Directorate (BİDEB).

## Conflicts of Interest

The authors declare no conflicts of interest.

## Supporting information


Data S1.

## Data Availability

We encourage all authors of articles published in MDPI journals to share their research data. In this section, please provide details regarding where data supporting reported results can be found, including links to publicly archived datasets analyzed or generated during the study. Where no new data were created, or where data is unavailable due to privacy or ethical restrictions, a statement is still required. Suggested Data Availability Statements are available in section “MDPI Research Data Policies” at https://www.mdpi.com/ethics.
