# Supplementary material for: The Effects of Tea Wastes Prepared Using Different Composting Methods on the Seedling Growth and Selected Biochemical Properties of Maize (Zea mays var. indurata)
Source: Food Sci Nutr. 2025 Jul 30;13(8):e70670. doi: 10.1002/fsn3.70670 (PMC12310293; doi:10.1002/fsn3.70670)
Supplement: Supplementary file 1 — Data S1. [file FSN3-13-e70670-s001.docx]

**Supplementary Materials
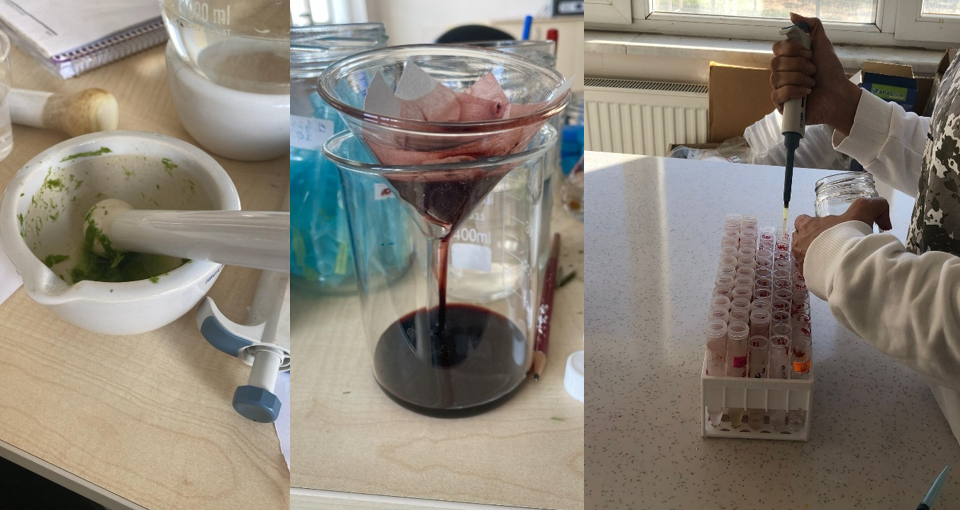
**

Figure A1. Grinding plant samples, performing pigment analyses, and reading in the spectrophotometera
